# Tumor invasion and metastasis regulated by microRNA-184 and microRNA-574-5p in small-cell lung cancer

**DOI:** 10.18632/oncotarget.6338

**Published:** 2015-11-16

**Authors:** Rui Zhou, Xiaoshu Zhou, Zhongyuan Yin, Jing Guo, Ting Hu, Shun Jiang, Li Liu, Xiaorong Dong, Sheng Zhang, Gang Wu

**Affiliations:** ^1^ Cancer Center, Union Hospital, Tongji Medical College, Huazhong University of Science and Technology, Wuhan, China; ^2^ Department of Oncology of The Affiliated Hospital of Qingdao University, Qingdao, China; ^3^ Department of Oncology, Second Xiangya Hospital of Central South University, Changsha, China

**Keywords:** miR-184, miR-574-5p, metastasis, prognosis, SCLC

## Abstract

Small-cell lung cancer (SCLC) is a highly aggressive neuroendocrine tumor that has an extremely poor clinical prognosis. Metastasis is the key event in SCLC progression, but its mechanism has not been fully elucidated. MicroRNAs (miRNAs) have been proven to participate in cancer processes, but their function in SCLC has not been thoroughly studied either. Here, we performed microarray and quantitative real-time PCR (qRT-PCR) analysesto identify the miRNAsassociated with metastasis and prognosis in SCLC as well as the correlation between serum and tissue. We also explored these miRNAs' promising molecular mechanisms by 3′UTR reporter assay and immunoblotting. We showed thatmiR-184 significantly attenuated the metastasis of SCLC, whereas miR-574–5p enhanced it. Both miRNAs were found to participate in β-catenin signaling by suppressing protein tyrosine phosphatase receptor type U (PTPRU)orendothelial PAS domain protein 1 (EPAS1). Furthermore, miR-574–5p was verified as an independent prognostic risk factor for SCLC. Taken together, our findings providea comprehensive analysis of the miRNA expression pattern in SCLC and indicate that miRNAs may serve as potential therapeutic and prognostic predictors in SCLC.

## INTRODUCTION

Small-cell lung cancer (SCLC) is a highly malignant cancer that accounts for 15–20% of lung cancers. SCLC originates from neuroendocrine cell precursors and is characterized by rapid growth and fatal metastasis [1]. Despite extreme sensitivity to chemotherapy and radiotherapy, 5-year survival rates remain at 5–10% [2]. The American Veterans Administration Lung Study Group (VALG) defines SCLC stages as limited disease (LD) and extensive disease (ED) based on whether the SCLC can be safely treated with definitive radiation doses. For LD patients, the median overall survival (mOS) duration is approximately 16–24 months, and the 5-year survival rate is 14% [3]. However, approximately 67% of newly diagnosed patients have ED, with median progression-free survival (mPFS) of 5.5 months and mOS of approximately 6–12 months [4, 5]. Therefore, a better understanding of the molecular mechanisms involved in SCLC progression is urgently needed.

MicroRNAs (miRNAs) are a class of conserved small non-coding RNAs that are widely found in nature. miRNAs may accelerate the degradation of or reduce the translation of their target mRNAs by binding to the 3′untranslated region (3′UTR) of target genes [6]. Abundant evidence indicates that miRNAs are involved in various fundamental biological processes, such as inflammation, cell cycle regulation, the stress response, differentiation, apoptosis and migration [7, 8]. Additionally, many miRNAs have been demonstrated to be oncogenes or tumor suppressors in different cancers profiled to date [9]. Serum miRNAs are resistant to harsh conditions, such as boiling, low/high pH, extended storage, and freeze-thaw cycles [10]. Therefore, stable, cell-free miRNAs in the serum have been extensively studied as potential biomarkers of cancer. Although many studies have examined the correlation between cancer and miRNAs, little is known about the function of miRNAs in SCLC, and especially metastatic process. Hence, this study attempted to explore the relationship between miRNAs and the malignant behavior of SCLC and may contribute to opening novel avenues for diagnosis and therapy.

Endothelial PAS domain protein 1 (EPAS1), also known as hypoxia-inducible factor 2 alpha subunit (HIF-2α), is a type of transcription factor that primarily induces the transcriptional response to hypoxia. Several lines of evidencehave indicated that EPAS1 is related to multiple aspects of cancers, including cell proliferation, angiogenesis, apoptosis, metabolism, metastasis and resistance to chemotherapy [11]. EPAS1 has also been reported to repress miR-15–16, leading to tumor angiogenesis and metastasis. In turn, down-regulation of miR-15–16 has been proven to be associated with a more advanced stage and a poor prognosis incolorectal carcinoma [12]. Meanwhile, protein tyrosine phosphatase receptor type U (PTPRU), also known as Purkinje cell protein 2 (PCP2), has been shown to function as a regulator of adhesion and proliferation in certain cancer cell types. However, the function of PTPRU varies between different cancers. PTPRU has beenidentified as an oncogene in gastric cancer [13] as well as in glioma [14], but it has also been confirmed as a tumor suppressor in colon cancer due to its dephosphorylation of β-catenin and inhibition of subsequent downstream signaling [15]. Zhao et al. also verified that PTPRU prevents tumor growth and the formation of metastases in breast cancer by attenuating tumor-associated angiogenesis and inducing the apoptosis and necrosis of tumor cells [16]. Thus, we focused on these two genes to clarify their interaction with miRNAs in SCLC.

Here, we compared serum miRNA expression profiles between LD and ED SCLC patients by microarray analysis. Furthermore, we verified these profiles in larger serum samples, tumor tissues and cell lines in attempt to discover miRNAs that increase or decrease the malignant behavior of SCLC. In this study, we showed that human miR-574–5p and miR-184 may be involved in the invasion and metastasis of SCLC *in vitro*. In addition, bioinformatics was used to investigate these miRNAs' target genes. We demonstrated that these miRNAs may alter the expression and function of β-catenin in H446 and H2227 cells by suppressing PTPRU and EPAS1. Clinically, we confirmed that high expression of miR-574–5p is associated with decreased PFS and OS in SCLC patients. Our findings also indicated that miR-574–5p and miR-184 are involved in SCLC migration and invasion processes, potentially serving as new prognostic factors and clinical therapeutic targets for SCLC.

## RESULTS

### Identification of serum miRNAs related to different stages of SCLC

Serum samples from a set of 72 SCLC patients (22 LD and 50 ED) were included in this study (Supplementary Table S1). For 45 patients, both serum and *in situ* tumor tissue were included. There were no significant differences in the distribution of age, gender, smoking status or Eastern Cooperative Oncology Group (ECOG) status between LD and ED patients, whereas the distribution of metastasis status did differ.

**Figure 1:**
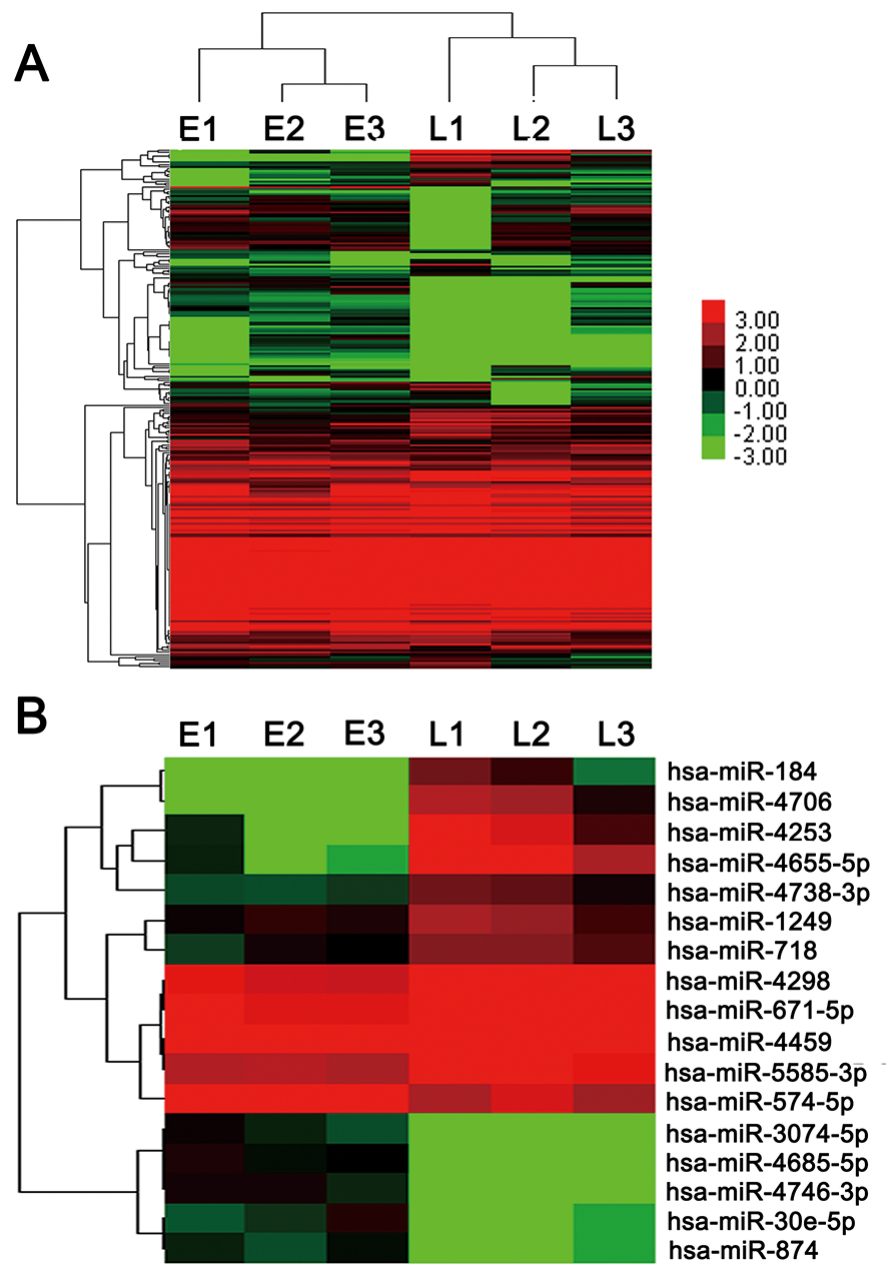
miRNA microarray of SCLC patients' serum samples **A.** Heat map of all miRNA expression differences between LD and ED SCLC serum samples included in the Sanger miRBase V18.0 database. **B.** Heat map summarizing the patterns of expression for 17 miRNAs whose expression was significantly (*p* <0.05 and foldchange > 2) altered in LD and ED SCLC serum samples.

To screen the metastasis-related miRNAs, we isolated total RNA from 3 ED-stage and 3 LD-stage patients' serum samples (Supplementary Table S2) and performed miRNA microarray analyses. As shown in Supplementary Table S3, we identified 6 miRNAs (hsa-miR-4685-5p, hsa-miR-4746-3p, hsa-miR-3074-5p, hsa-miR-30e-5p, hsa-miR-874 and hsa-miR-574-5p) overexpressed in ED compared with LD. Meanwhile, 11 miRNAs (hsa-miR-4706, hsa-miR-184, hsa-miR-4253, hsa-miR-4655-5p, hsa-miR-4298, hsa-miR-671-5p, hsa-miR-4459, hsa-miR-4738-3p, hsa-miR-718, hsa-miR-1249 and hsa-miR-5585-3p) were down-regulated. The unsupervised hierarchical clustering of the 250 miRNAs with acceptable detection intensities is shown in Figure 1A. The heat map of the 17 miRNAs (Figure 1B) demonstrated the differential expression signatures between LD and ED SCLC patients.

**Figure 2:**
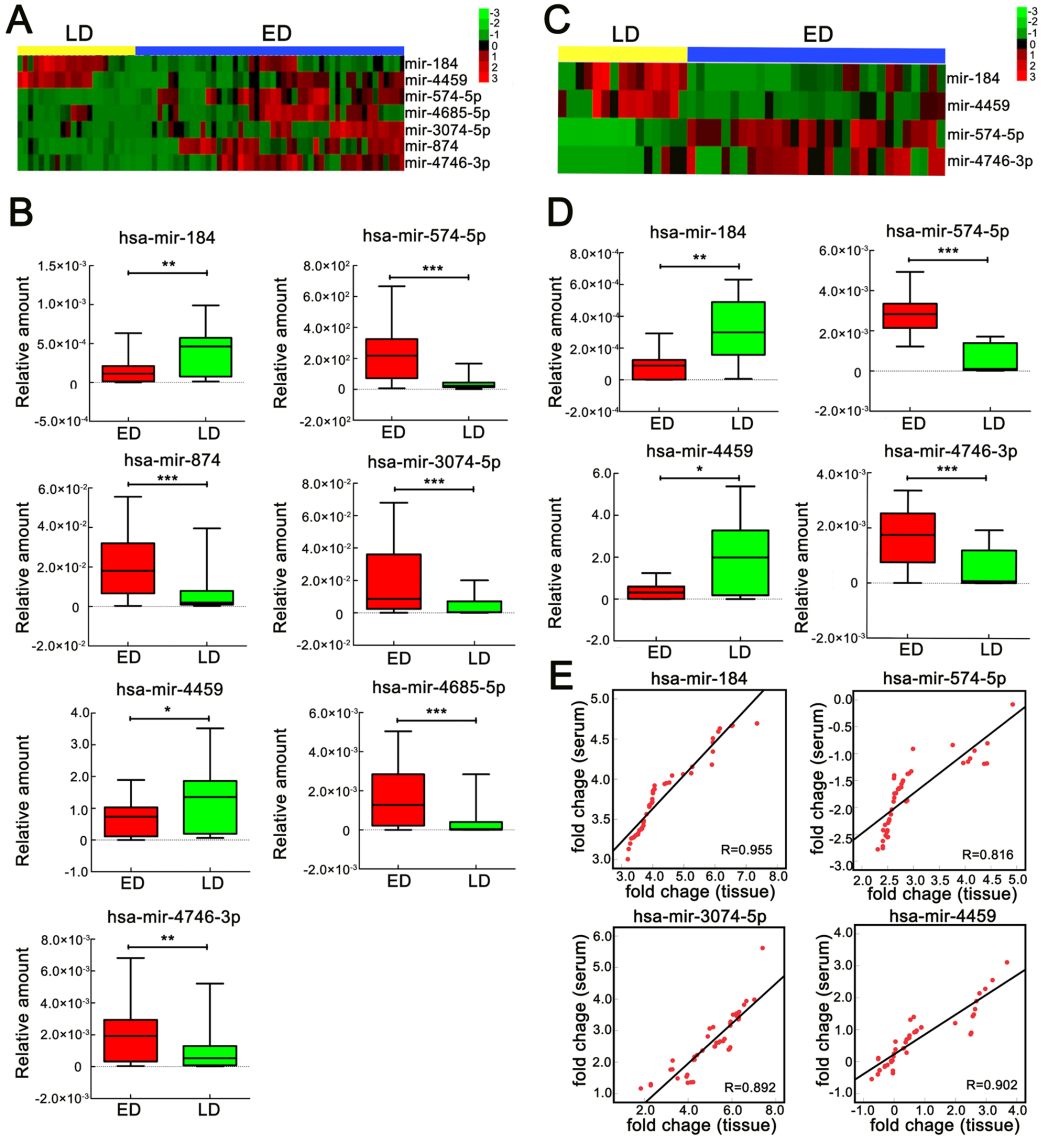
Significantly differentially expressed miRNAs in serum and tissue between ED and LD SCLC patients **A.** Heat map of 7 miRNAs whose expression was significantly (*p* <0.05) altered between ED (blue bar, *n* = 50) and LD (yellow bar, *n* = 22) SCLC patients' serum samples, as measured by qRT-PCR. **B.** qRT-PCR validation of significant differentially expressed miRNAs in serum samples, as analyzed using the Mann-Whitney U test. **C.** Heat map of 4 miRNAs whose expression was significantly (*p* <0.05) altered between ED (blue bar, *n* = 30) and LD (yellow bar, *n* = 15) SCLC patients' tissue samples, as measured by qRT-PCR. **D.** qRT-PCR of significant differentially expressed serum miRNAs in tissue samples, as analyzed using the Mann-Whitney U test. **E.** Pearson's correlation scatter plot of miRNA levels in matched SCLC samples. *, *p* < 0.05; **, *p* < 0.01; ***, *p* < 0.001. Red, ED, Extensive disease; Green, LD, Limited disease.

We next detected the expression of 17 candidate miRNAs selected from the initial screening using individual qRT-PCR assays. In the initial pilot trial, we tested the relative abundance of the miRNAs, and 15 of the 17 yielded acceptable and consistent signals (data not shown). Therefore, these miRNAswere chosen for the subsequent confirmation study. We next performed qRT-PCR on the 15 miRNAs in the validation cohort (22 LD and 50 ED). In total, 7 miRNAs were significantly correlated with SCLC metastasis (Figure 2A). Of these 7 miRNAs, 5 (miR-574-5p, miR-874, miR-3074-5p, miR-4685-5p and miR-4746-3p) were overexpressed in ED, whereas 2 (miR-184 and miR-4459) were down-regulated (Supplementary Table S4). The boxplot diagram revealed the relationship between the 7 miRNAs and the stages more clearly (Figure 2B).

### Correlation of miRNA expression between matching tissue and serum samples

To determine the correlation of miRNAs between tissue and serum samples, we investigated the expression of the selected 7 miRNAs in 45 matching tissue and serum samples (Supplementary Table S5). The results showed that miR-184 (*p* <0.001), miR-574-5p (*p* <0.001), miR-3074-3p (*p* <0.001) and miR-4459 (*p* <0.001) had significant correlation expression profiles (Figure 2E), which suggested that these 4 miRNAs may reflect most of the characteristic expression patterns of their tissue counterparts. Consequently, we next compared the tissue miRNA expression between LD and ED. The result showed that miR-184, miR-574-5p, miR-4459 and miR-4746-3p were significantly associated with the disease stage (Figure 2D, Supplementary Table S6). The heat map and the scatter plot showed the differential miRNA expression between LD and ED SCLC patients more clearly (Figure 2C).

### Clinical importance of miR-574-5p as an independent prognostic factor for PFS/OS in SCLC

**Figure 3:**
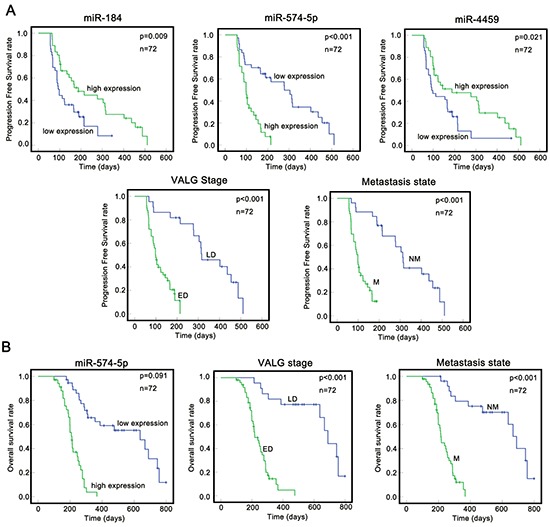
Correlation between the expression of miRNAs/clinical features and prognosisin SCLC **A.** A K-M analysis indicated high expression of miR-574-5p (*p* <0.001). ED (*p* <0.001) and the metastasis state (*p* <0.001) were significantly associated with poorer PFS among SCLC patients. **B.** A K-M analysis indicated that high expression of miR-574–5p (*p* <0.001), ED (*p* <0.001) and the metastasis state (*p* <0.001) were significantly associated with poorer OS among SCLC patients. ED, Extensive disease; Green, LD, Limited disease. M, metastasis state; NM, no-metastasis state.

To determine whether the 7 metastasis-related miRNAs are associated with PFS and OS, we investigated the same 72 SCLC patients, whose median follow-up time was 256.5 days. During the follow-up period, 57 patients (79.1%) exhibited disease progression, 15 patients (20.9%) were lost to follow-up,and 50 (69.4%) died from SCLC. We next performed a Kaplan-Meier (K-M) analysis (log-rank test) and multiple Cox proportional hazard regression analysis to determine whether these miRNA predictors were confounded by underlying clinical conditions. The K-M analysis revealed that miR-574-5p, metastasis and ED were prognostic risk factors for PFS, whereas miR-184 and miR-4459 were functionally opposite (Figure 3A, Table 1). The multivariate survival model, which controlled for potential confounding covariates, demonstrated that miR-574-5p (*p* = 0.001) and metastasis (*p* <0.001) increased the risk of SCLC progression (Table 1). For OS analysis, K-M analysis showed that miR-574-5p, metastasis and ED were prognostic risk factors for OS (Figure 3B, Table 2). However, multivariate Cox regression analysis demonstrated that only miR-574-5p (*p* <0.001) and metastasis (*p* <0.001), and not the VALG stage, were independent risk factors for OS in SCLC (Table 2). Taking these clinical results together, we conclude that only miR-574-5p is anindependent prognostic predictor in SCLC.

**Table 1:** Multivariate cox regression analysis of factors associated with PFS

Variable	Log-Rank Analysis^a^	Multivariate Analysis^b^		
	*p*-value	OR	95%CI	*p*-value
Gender (F vs M)	0.603			
Smoking status (Yes vs No)	0.280			
Age (>57yearsvs ≤ 57years)	0.627			
ECOG status (0 vs 1)	0.693			
VALG stage (LD vs ED)	< 0.001			
**Metastasis**	< 0.001	0.139	(0.05, 0.386)	< 0.001
**hsa-mir184**	0.009	2.023	(1.131, 3.618)	0.017
**hsa-mir574-5p**	< 0.001	0.304	(0.150, 0.618)	0.001
hsa-mir4459	0.021			

NOTE: Bold indicates significant values in the multivariate analysis.

a Log-rank test based on Kaplan-Meier analysis

b Multivariate analysis, Cox proportional hazards regression

Abbreviations: F, female; M, male. ED, extensive disease; LD, limited disease.

**Table 2:** Multivariate cox regression analysis of factors associated with OS

Variable	Log-Rank Analysis^a^	Multivariate Analysis^b^		
	*p*-value	OR	95%CI	p-value
Gender (F vs M)	0.566			
Smoking status (Yes vs No)	0.097			
Age (>57yearsvs ≤ 57years)	0.360			
ECOG status (0 vs 1)	0.255			
VALG stage (LD vs ED)	< 0.001			
**Metastasis**	< 0.001	0.149	(0.052, 0.424)	< 0.001
**hsa-mir574-5p**	< 0.001	0.288	(0.138, 0.601)	< 0.001

NOTE: Bold indicates significant values in the multivariate analysis.

a Log-rank test based on Kaplan-Meier analysis

b Multivariate analysis, Cox proportional hazards regression

Abbreviations: F, female; M, male. ED, extensive disease; LD, limited disease.

### Candidate miRNA transfection into SCLC cell lines H446 and H2227

Cell proliferation, migration, invasion and angiogenesis are among the common functions required by tumor cells for metastatic progression in target microenvironments. In the present study, H446 and H2227 cells were chosen to represent the typical SCLC cell lines. To investigate the biological effects of the 7 candidate miRNAs on the invasiveness of SCLC, *in vitro* functional analyses were performed using overexpression and inhibition strategies based on miRNA mimics and inhibitors, respectively, transfected into H446 and H2227 cells. The qRT-PCR results revealed that the miRNAs were markedly overexpressed or significantly inhibited after 72 hours of treatment with the transfection mimics and inhibitors, respectively (Supplementary Figure S1).

### miR-574-5p promotes and miR-184 suppresses SCLC metastasis and invasion *in vitro*

**Figure 4:**
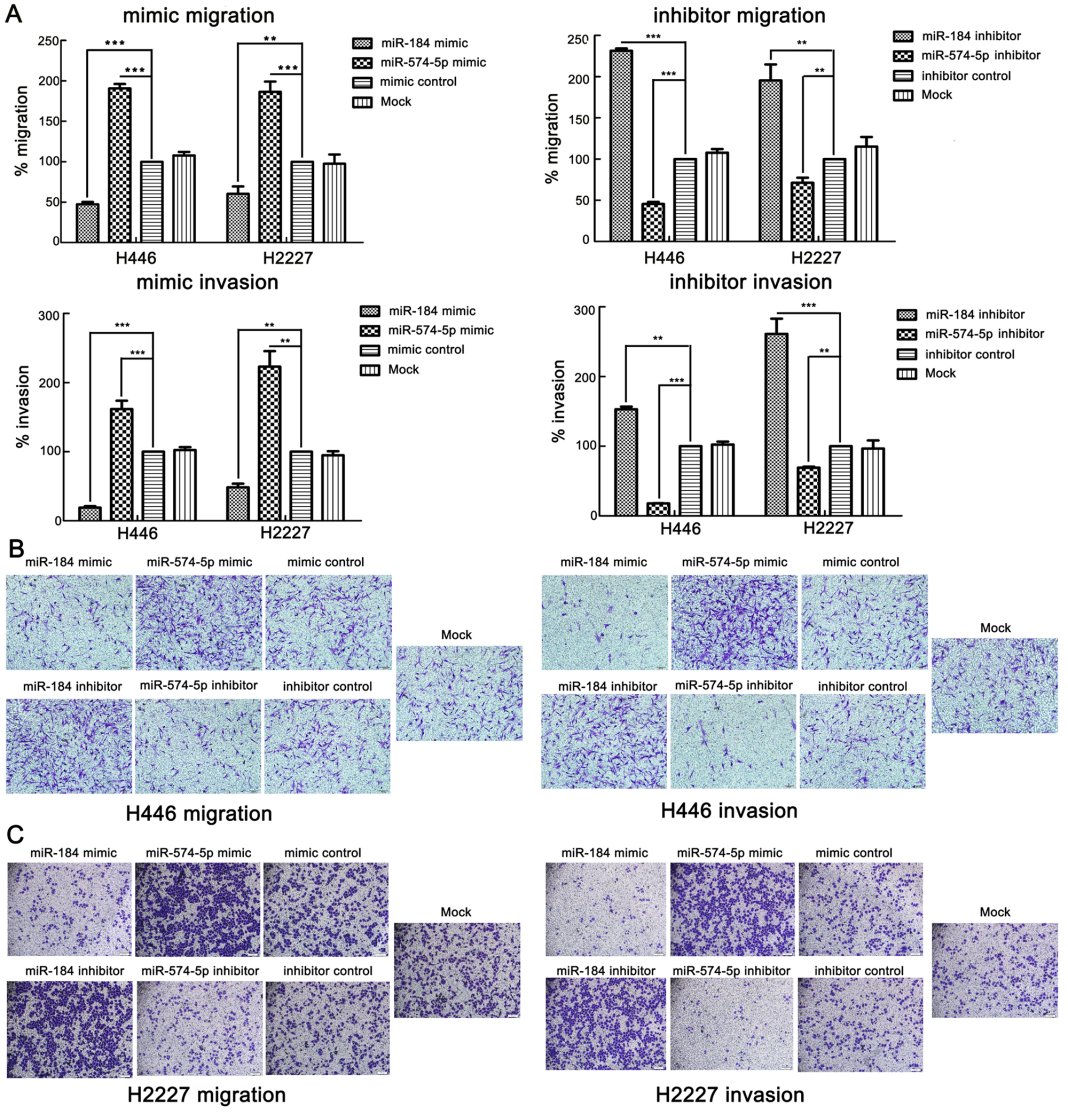
miR-574-5p promotes the metastasis and invasion of SCLC cell lines, whereas miR-184 suppresses it **A.** Upper panel, statistical results for transwell migration assays of H446 and H2227 cells transfected with the indicated mimics or inhibitors (analyzed by *t*-test). Lower panel, statistical results for transwell invasion assays of H446 and H2227 cells transfected with the indicated mimics or inhibitors (analyzed by *t*-test).% migration=[mean number of cells invading the membrane/mean number of cells migrating through the control insert membrane] × 100.% invasion=[mean number of cells invading the Matrigel membrane/mean number of cells migrating through the control insert membrane] × 100. **B.** Images from transwell migration assays and invasion assays of H446 cells (×100). **C.** Images from transwell migration assays and invasion assays of H2227 cells (×100).*, *p* < 0.05; **, *p* < 0.01; ***, *p* < 0.001.

The above clinical findings suggest that the 7 candidate miRNAs may participate in SCLC progression. To evaluate which one actually increases or decreasesthe metastatic ability of SCLC, we first conducteda wound healing assay to screen these candidate miRNAs. The assay indicated that miR-574-5p enhanced the migration ability of H446 cells. In contrast, miR-184 repressed this ability (Supplementary Figure S2). We then used transwell assays to verify this finding. Not surprisingly, the transwell assays, including migration and Matrigel invasion assays, all confirmed that miR-574-5p promoted the metastatic ability of the SCLC cell lines, whereas miR-184 repressed this ability (Figure 4).

### miR-574-5p represses PTPRU, and miR-184 represses EPAS1

**Figure 5:**
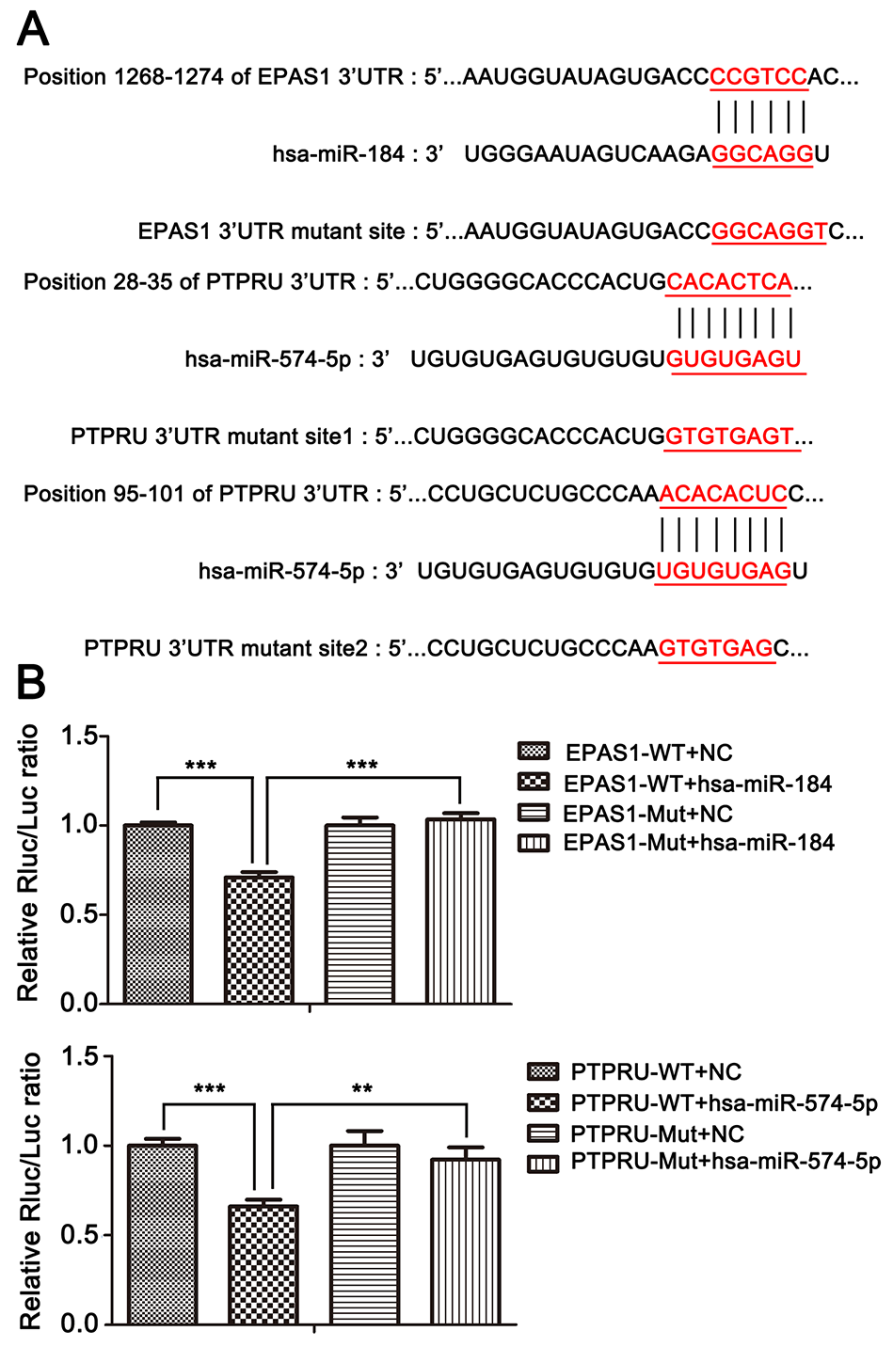
miR-574-5p and miR-184 directly suppress PTPRU and EPAS1, respectively, and can alternately activate or inhibit β-catenin signaling **A.** Upper panel, sequence of miR-184, wild-type and mutant 3′UTR of EPAS1. Lower panel, sequence of miR-574-5p, wild-type and mutant 3′UTR of PTPRU. **B.** Upper panel, luciferase assay of HEK293T cells co-transfected with an miR-184 mimic and firefly luciferase vector containing the 3′UTR or mutant 3′UTR of EPAS1. Lower panel, luciferase assay of HEK293T cells co-transfected with an miR-574-5p mimic and firefly luciferase vector containing the 3′UTR or mutant 3′UTR of PTPRU.*, *p* < 0.05; **, *p* < 0.01; ***, *p* < 0.001.

**Figure 6:**
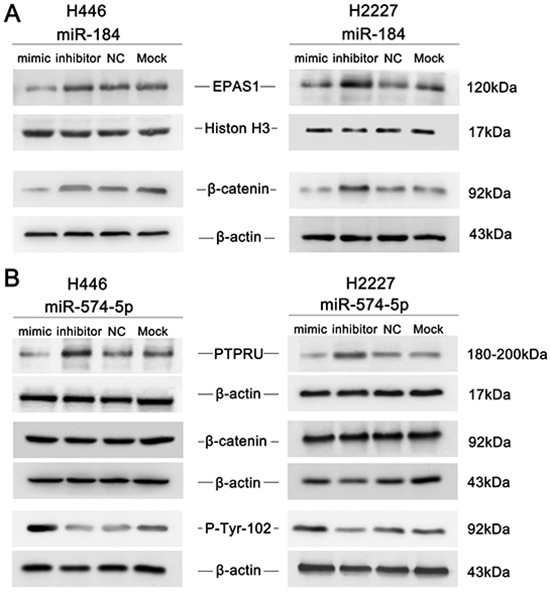
An immunoblot analysis was performed to determine the expression levels of EPAS1, PTPRU and β-catenin in response to miR-184 or miR-574-5p in SCLC cell lines **A.** miR-184 overexpression inhibited EPAS1 and β-catenin in H446 and H2227 cells, whereas miR-184 inhibition had the opposite effects. **B.** miR-574-5p overexpression inhibited PTPRU and enhanced the tyrosine phosphorylation of β-catenin in H446 and H2227 cells, whereas miR-574-5p inhibition had the opposite effects.

To determine the mechanisms of the functions of miR-574-5p and miR-184 in SCLC invasion and metastasis, the target prediction programs miRDB, miRanda and TargetScan were used to search for these miRNAs' predicted direct target genes. Among the numerous targets predicted, 2 genes, namely, PTPRU and EPAS1,were previously reported to be involvedin cancer metastasis [17]. Bioinformatics analysis of the 3′UTRs of EPAS1 and PTPRU showed that at least 6 nucleotides of the EPAS1 sequence are complementary to miR-184 and that at least 2 different parts of the PTPRU sequence are complementary to miR-574-5p (Figure 5A). To determine whether miR-184 and miR-574-5p increase or decrease SCLC metastasis through a combination of their predicted genes' 3′UTRs, we cloned the 3′UTRs of EPAS1 and PTPRU downstream of the luciferase gene in 293T cells as a reporter assay. A dual luciferase experiment showed that the introduction of miR-184 markedly suppressed the expression of the luciferase gene containing the 3′UTR of EPAS1 and that miR-574-5p suppressed the expression of the luciferase gene containing the 3′UTR of PTPRU. However, the luciferase activities of the reporter constructs mutated at the combination site were unaffected (Figure 5B). To experimentally verify whether these two target genes can be functionally suppressedby miR-184 or miR-574-5p, cells were transfected with the corresponding mimic or inhibitor, and the protein levels were assessed by immunoblotting. The results showed that miR-184 attenuated EPAS1 expression (Figure 6A) while miR-574-5p suppressed PTPRU (Figure 6B) in SCLC cell lines.

The above data suggest that EPAS1 is the functional target of miR-184 and that PTPRU is the functional target of miR-574-5p.

### miR-574-5p and miR-184 alter β-catenin signaling

PTPRU has been reported [15, 18] to inhibit cell growth and invasion by decreasing β-catenin tyrosine phosphorylation. Because the presence of phosphorylated tyrosine residues on β-catenin is correlated with loss of E-cadherin-mediated cell adhesion, it was determined that PTPRU-mediated dephosphorylation of β-catenin contributes to enhanced cell adhesion [15]. Furthermore, EPAS1 has been reported to enhance the activity of β-catenin as a transcription factor [17, 19]. Therefore, we wondered whether miR-574-5p and miR-184 increase or decrease β-catenin expression in SCLC. As shown in Figure 6, transfection with miR-574-5p enhanced thetyrosine phosphorylation of β-catenin, and inhibition of miR-184 increased the total β-catenin expression in SCLC cell lines. Consequently, overexpression of miR-574-5p and inhibition of miR-184 can both activate the β-catenin signaling pathway.

Taking these clinical results together with the findings of the aforementioned experimental studies, we conclude that miR-184 attenuates the metastasis of SCLC, whereas miR-574-5p has the opposite effect. The β-catenin signaling pathway may have an important function in this process.

## DISCUSSION

SCLC is a highly aggressive disease whose clinical prognosis mainly depends on the stage. However, our understanding of the molecular mechanism of SCLC is limited. miRNAs have been confirmed to participate in the development of numerous tumor types in recent years, and especially tumor metastasis [20]. Although there are numerous studies regarding miRNAs, few have examined the association of miRNA expression with SCLC, particularly in clinical samples. Therefore, we screened miRNA expression in clinical specimens of SCLC to identify specific miRNA candidates that characterize different SCLC stages and also analyzed the mechanism of these miRNAs' biological behavior.

Endogenous circulating miRNAs have attracted significant attention regarding the diagnosis, prognosis and metastasis of cancer [21]. In fact, numerous studies have been performed to assess the relationship between serum miRNAs and cancer [22, 23]. Using microarray analysis, we demonstrated that serum from ED-SCLC has a significantly different miRNA expression profile compared with serum from LD-SCLC. Seven miRNAs were confirmed to be expressed differentially in the serum, as assessed by qRT-PCR, which implied that systematic changes in miRNA expression occur during the tumorigenesis of SCLC and may be involved in systemic regulation. Additional studies showed that the expression levels of 4 miRNAs (miR-184, miR-574-5p, miR-4459, miR-4746-3p) are positively correlated between serum and tissue and that the expression of miR-184, miR-574-5p, miR-3074-5p and miR-4459 in tissue is relevant to the stage of SCLC. Wang et al. demonstrated a high correlation of miRNA expression levels between breast tumor tissues and serum, so selective expression and modulation of miRNAs could be potential blood-based biomarkers for breast cancer diagnosis, grading and prognosis [24]. In a study of patients with prostate cancer, Brase et al. demonstrated that miR-375 and miR-141 were highly expressed in all samples and were significantly up-regulated in tumors compared with normal tissues. Furthermore, these miRNAs' levels were correlated with a high Gleason score and a lymph-node-positive status in a second, independent validation study [25]. The above studies indicate that certain circulating miRNAs may be derived from tumor cells and that these miRNAs can directly increase or decrease the malignant behavior of tumor cells. A change in these miRNAs' expression can thus reflect tumor progression.

miRNAs have been extensively investigated as prognostic factors [10, 26]. Serum miRNAs are more favorable for this purpose because serum detection methods are simpler and more efficient [27, 28]. Another important discovery in the present study is that high expression of miR-574-5p was an independent prognostic risk factor in patients with SCLC, as determined by multivariate Cox regression of PFS and OS. miR-574-5p has been reported to be an oncogene in various types of cancer. In particular, this miRNA has been shown to be up-regulated in esophageal squamous cell carcinoma [29] and colorectal cancer [30] tumor tissues compared with adjacent non-tumor tissues. Additionally, miR-574-5p was found to be significantly increased in early-stage NSCLC serum samples with respect to controls, making it a reliable serum-based biomarker [31]. Previous research demonstrated that miR-574-5p was significantly associated with chemoresistance in SCLC but did not contribute significantly to survival as an independent factor [32]. Because our study is a prospective study, in contrast to the retrospective study mentioned above, our results are different but not inconsistent. Overall, these results verify that high expression levels of miR-574-5p in serum may be indicative of a poor prognosis in patients with SCLC.

miRNA expression differences in serum do not necessarily alterthe function of tumor cells. Therefore, we further investigated the biological function of the candidate miRNAs *in vitro*. Interestingly, miR-574-5p can also function as an oncogene to facilitate the invasion and migration of the SCLC cell linesH446 and H2227. Similarly, miR-574-5p has been shown to greatly enhance the migration and invasion of colorectal cancer [30] and large-cell lung cancer [33]. Although the targets were different, these results support our findings that miR-574-5p functions as an oncogene that is expressed in a variety of tumor tissues. Thus, this miRNA may form the basis of new approaches for cancer therapy via the mechanisms of its regulation of tumors.

Additionally, we found that miR-184 inhibits cell migration and invasion *in vitro*. In contrast to the clear oncogene effect of miR-574-5p, miR-184 has a more complicated function in cancer: it may be oncogenic in squamous cell carcinoma of the tongue [34, 35] and in hepatocellular carcinoma [36], but it may also be involved in inhibiting cell growth in neuroblastoma [37], nasopharyngeal carcinoma [38] and non-small-cell lung cancers [39]. Although the function of miR-184 in tumorigenesis remains unclear, here, we demonstrated that miR-184 functioned mainly as a tumor suppressor in SCLC.

The mechanism by which miRNAs altergene expression remains controversial, but most studies have suggested that miRNAs are primarily processed by the RNA-mediated interference machinery to trigger partial or complete target gene mRNA degradation [40]. Our bioinformatics analysis revealed that miR-574-5p could bind the 3′UTR of PTPRU, and we observed that the expression of PTPRU could be repressed by miR-574-5p. Furthermore, we found that miR-574-5p could promote the tyrosine phosphorylation of β-catenin *in vitro*. PTPRU is a novel member of the receptor protein tyrosine phosphatase (R-PTP) family. The potential participation of PTPRU in cell-cell recognition and adhesion is supported by its co-localization with cell adhesion molecules, such as catenin and E-cadherin, at sites of cell-cell contact [41]. Yan et al. claimed that tyrosine phosphorylation of β-catenin leads to greater stimulation of β-catenin-Tcf-mediated transcription and that PTPRU-mediated dephosphorylation of β-catenin is necessary for PTPRU to inhibit β-catenin. PTPRU specifically acted as an inhibitor of metastasis, which led to increased membrane-bound E-cadherin and greater stabilization of adherens junctions by dephosphorylation of β-catenin [42]. Thus, repressed expression of PTPRU may cause increased mobility among cancer cells. PTPRU was also reported to be significant down-regulatedin lung cancer cell lines [43]. We therefore postulate that miR-574-5p promotes SCLC metastasis by repressing PTPRU and ultimatelyincreases the tyrosine phosphorylation of β-catenin and reduces intercellular adhesion.

Bioinformatics analysis also showed that miR-184 could post-transcriptionally down-regulate its target EPAS1 by directly bindingthe 3′UTR. EPAS1, also known as HIF-2α, is a transcription factor that is directly involved in various tumor types [44]. Research by Kim et al. causally implicated HIF-2α in the pathogenesis of lung cancer in mice,demonstrated that HIF-2α can promote the expression of markers of the epithelial-mesenchymal transition (EMT)*in vivo*, and defined HIF-2α as a promoter of tumor growth and progression. This research further suggested a possible causal relationship between HIF-2α and prognosis in patients with NSCLC [45]. In research by Choi et al., HIF-2α was found to be required for β-catenin activation and for proliferationin cells [46]. In our study, it was observed that miR-184 can directly inhibit synthesis of the HIF-2α proteinin H446 and H2227 cells, in turn causing a decline in β-catenin expression.

β-catenin is involved in many biological processes; the classic one is the Wnt/β-catenin pathway [47]. It has also been confirmed that β-catenin is related to the E-cadherin pathway [48]. Interestingly, β-catenin may stimulate or inhibit the two pathways at the same time, impacting intercellular adhesion and invasion abilities and thus the processes of tumor invasion and metastasis [49]. A study by Saydam et al. has demonstrated that restraining miR-200a in meningioma could inhibit the function of E-cadherin and activate the Wnt/β-catenin pathway at the same time to promote tumor growth [50].

In summary, our research is the first report to unveil serum miR-574-5p as a novel prognostic biomarker of PFS/OS in SCLC. Forced expression of miR-574-5p promotes SCLC cell line invasion and metastasis, whereas miR-184 has the reverse effect. These miRNAs can directly suppress PTPRU or EPAS1 and may be involved in β-catenin signaling in this way. Suppression of SCLC cell migration by miRNAs indicates that such a strategy may serve as a basis for the development of therapies against metastasis.

## MATERIALS AND METHODS

### Patients and clinical samples

This study was conducted in 72 SCLC patients with an ECOG performance status of 0–1 and from Wuhan Union Hospital (Huazhong University of Science and Technology, China) between November 2012 and June 2014. All samples were collected from consenting individuals according to theprotocols approved by the Ethics Review Board of Wuhan Union Hospital, Huazhong University of Science and Technology. None of the patients had undergone treatment before enrollment. All participants underwent strict imaging and physical examinations as well as collection of their history, including demographic characteristics and medical and smoking histories. All of the enrolled patients were diagnosed by pathology and staged by specialized oncologists via VALG staging. After hospital admission, a 5 ml peripheral blood sample was drawn into a gold-top serum-separating tube, processed for serum extraction within 2 hours, and then placed at −80°C for long-term storage. In total, 45 patients had their tumor tissuesbiopsied by CT-guided percutaneous lung biopsy, which yielded approximately 1×0.2 cm biopsy samples that were then soaked in 1.5 ml RNALatersolution(Ambion,AM7021) and subsequently stored at −80°C. Serum and tissue samples were selected retrospectively at the time of analysis according to the following requirements: 1. The patient had been diagnosed with SCLC; 2. A sufficient volume of serum was available for RNA isolation; and 3. Demographic, clinical, and follow-up data were available for the patient. All patients received the standard treatments recommended by the National Comprehensive Cancer Network (NCCN) guideline for SCLC.

### miRNA microarray

Total RNA was isolated from 1 mlserum using anmiRNeasy Mini Kit (Qiagen, 217004) following the manufacturer's protocol. Total RNA (200ng) was dephosphorylated and labeled with Cyanine3-pCp, without fractionation or amplification, using an Agilent miRNA Complete Labeling and Hyb Kit (p/n5190–0456) following the manufacturer's instructions. An Agilent Human miRNA 8 × 60 Kit, containing 1887 mature human miRNA sequences, was used for gene expression analysis, and an Agilent SureScan Microarray Scanner (G2565CA) was used to collect the signals. Agilent Feature Extraction (v10.7) and Agilent GeneSpring software were used to analyze the resulting signals. Agilent Labeling Spike-In RNA and HybSpike-In RNA were used for quality control in all processes. A list of miRNAs contained in the array is available from the Sanger miRBase V18.0 database.

### Microarray data

The data discussed in this publication have been deposited in the NCBI's Gene Expression Omnibus and are accessible through GEO Series accession number GSE67804 (http://www.ncbi.nlm.nih.gov/geo/query/acc.cgi?acc=GSE67804).

### Total RNA extraction

Total RNA was isolated from 400μlserum samples using the mirVana™ PARIS™ Kit (Applied Biosystems, AM1556) following the manufacturer's protocol. A total of 25 fmol synthetic *C.elegans* miRNA (cel-miR-39-3p, Qiagen) was introduced after the addition of denaturing solution to each sample to monitortechnical variations in RNA extraction, as has been described before [51]. Total RNA was isolated from cells and tissues using an E.Z.N.A.™ Total RNA Kit II (OMEGA R6934–02) following the manufacturer's protocol.

### Reverse transcription PCR and qRT-PCR

Each miRNA was specifically reverse transcribed according to the manufacturer's protocol using the TaqManMicroRNA Reverse Transcription Kit with stem-loop RTprimer and the TaqMan^®^MicroRNA Reverse Transcription Kit (Applied Biosystems, 4366596). For real-time PCR, 2μl diluted reverse transcription product wasmixed with 10μl SYBR Select Master Mix(2×), 0.8μl forward and reverse primers and 6.4μl nuclease-free water to a final volume of 20μl (Applied Biosystems, SYBR Select Master Mix,4472908). All reactionswere performed in triplicate on a StepOnePlusReal-Time PCR System (Applied Biosystems) under the following conditions: 50°C for 2 min and 95°C for 2 min, followed by 40 cycles at 95°C for 3 s and 60°C for 30 s. The TaqMan stem-loop primer for reverse transcription PCR and the forward and reverse primers for real-time PCR are shown in Supplementary Table S1.

### Cells and cell culture

The human SCLC cell lines H446 and H2227 and the HEK293T cell line were purchased from the Cell Resource Center, Shanghai Institute of Biochemistry and Cell Biology, Chinese Academy of Sciences. The H446 and HEK293T cells were maintained at 37°C in a humidified air atmosphere containing 5% carbon dioxide and cultured in RPMI 1640 (HyClone, SH30809.01B) containing 15% heat-inactivated fetal bovine serum (Gibco,16000-044). The H2227 cells were maintained at 37°C in a humidified air atmosphere containing 5% carbon dioxide and cultured in DMEM:F12 Medium (HyClone,SH30023.01B) containing 5% heat-inactivated fetal bovine serum (Gibco,16000-044), 0.005 mg/ml insulin (Sigma, I5500), 0.01 mg/ml transferrin (Sigma, T3309), 30nMsodium selenite (final concentration, Sigma, 214485), 10 nMhydrocortisone (final concentration, Sigma, H0008), 10 nMβ-estradiol (final concentration, Sigma, E8875), and extra 2 mM L-glutamine (final concentration of 4.5 mM, Sigma, G7513).

### Mimics and inhibitors for transfection

All mimics and inhibitors were designed and constructed by RiboBio, Guangdong, China. Cells were transfected with mimics and inhibitors using Lipofectamine 2000 (Invitrogen,11668–019) following the manufacturer's protocol. The final concentration of the mimics was 50 nM, and that of the inhibitors was 100 nM. The cells were subjected to further experimentation at least 48 hours after transfection. All mimics' and inhibitors' transfection efficiencies have been verified (Supplementary Figure S1).

### 3′UTR reporter assay

HEK293T cells were seeded into 96-well plates at 15,000 cells per well the day before transfection. A mixture of 5 fmol miRNA mimic and 100ng wild-type pmiR-RB-REPORT™-hRluc-3′UTR or mutant pmiR-RB-REPORT™-hRluc-3′UTR was transfected into HEK293T cells by addingLipofectamine^®^ 2000 (Invitrogen, 11668-500) to each well. After 6 hours, the medium was replaced with complete medium. Luciferase activities were measured 48 hours later with a Dual-Glo^®^ Luciferase Assay System (Promega, E1910). The hluc luciferase activities were used as an internal control for transection efficiency. The wild-type pmiR-RB-REPORT™-hRluc-3′UTR and mutant pmiR-RB-REPORT™-hRluc-3′UTRof EPAS1 and PTPRU were purchased from RiboBio, Guangdong, China. The sequences are shown in Supplementary Sequences S1–2.

### Wound healing assay

Approximately 5 × 10^5^ H446 cells suspended in 2 ml complete medium were plated in 6-well plates and cultured at 37°C for approximately 24 hours. Once monolayers of cells had formed, wounds were generated by scraping with a200μlplastic pipette tip. The monolayers rinsed several times with medium to remove dislodged cells, after which culture was continued in 2% serum at 37°C for 12 or 24 hours in an incubator containing 5% CO_2_. Cells that had migrated into the wound area were photographed using an inverted microscope at 100x magnifications (Olympus, CKX41). The distance was measured using ImageJ2x.

### *In vitro* migration and invasion assays

For the transwell migration assay, 2 × 10^4^/200μl H446 or H2227 cells were plated in the top chamber with the non-coated membrane (24-well insert, pore size: 8 μm, BD Biosciences). For the invasion assay, 5 × 10^4^/200μlH446or H2227 cells were plated in the top chamber with extracellular matrix gel(Sigma, E1270)-coated membranes (24-well insert, pore size: 8 μm, BD Biosciences). In both assays, the cells were plated in medium without serum, and medium with 15% serum was used as a chemoattractant in the lower chamber. The cells were incubated at 37°C for 24 hours, and cells that did not migrate through or invade the pores were removed with a cotton swab. Cells on the lower surface of the membrane were fixed with methanol and stained with0.1% crystal violet (Sigma). Cells in 9 random fields of view at100xmagnification were counted and expressed as the average number of cells per field of view. Pictures were taken with an inverted phase-contrast microscope at 100x magnification (Olympus,CKX41).

### Western blot analysis

Western blot analysis was performed according to standard procedures, as previously described [52]. The following primary antibodies were used: anti-PTPRU mAb (1:1000, R&D Systems^®^ MAB7475), anti-EPAS1mAb (1:1000, CST 7096S), anti-β-catenin mAb (1:2000, CST 8480S), anti-P-Tyr-102 mAb (1:2000, CST 9416S), anti-β-actin mAb (1:2000, Santa Cruz sc-47778) andanti-H-histone 3mAb (1:1000, Santa Cruz sc-134355). The following secondary antibodies were used: peroxidase-conjugated Affinipure Goat Anti-Mouse IgG(H+L) (1:3000, Proteintech SA00001-1) and peroxidase-conjugated Affinipure Goat Anti-Rabbit IgG(H+L) (1:3000, Proteintech SA00001-2).

### Statistical analysis

All statistical analyses were conducted using SPSS 21.0 statistical software. The Mann-Whitney U test was used to determine the significance of differences between the expression levels of miRNAs in the serum or tissue of LD and ED patients. The correlation of serum and tissue miRNA expression was determined using Pearson correlation coefficients in a two-tailed test. The log-rank test (based on K-M analysis)and Cox proportional hazards regression was used to analyze the effect of clinical variables and miRNAs onpatients' PFS and OS. Continuous data were compared using Student's 2-tailed *t*-test. In all cases, *p* < 0.05 was considered statistically significant.

## SUPPLEMENTARY FIGURES, TABLES AND SEQUENCES


