# Supplementary material for: Tumor invasion and metastasis regulated by microRNA-184 and microRNA-574-5p in small-cell lung cancer
Source: Oncotarget. 2015 Nov 16;6(42):44609–22. doi: 10.18632/oncotarget.6338 (PMC4792579; doi:10.18632/oncotarget.6338)
Supplement: Supplementary file 1 [file oncotarget-06-44609-s001.pdf]

SUPPLEMENTARY FIGURES, TABLES AND SEQUENCES

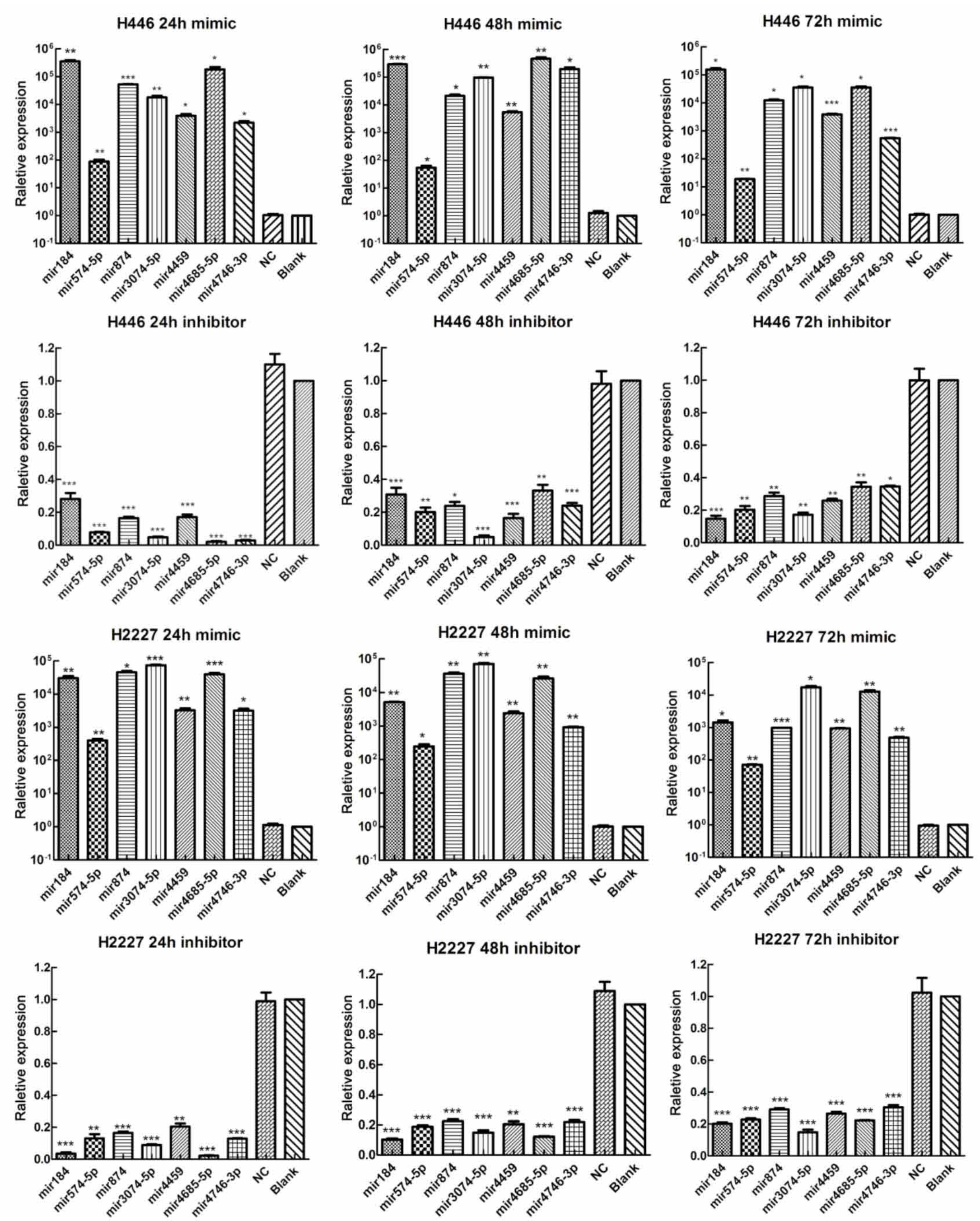

Supplementary Figure S1: miRNAs were markedly overexpressed or significantly inhibited after 72 hours of treatment with transfection mimics and inhibitors, respectively, of H446 and H2227 cells compared with the NC and blank control groups, as validated by qRT-PCR. *, $p < 0.05$; **, $p < 0.01$; ***, $p < 0.001$.

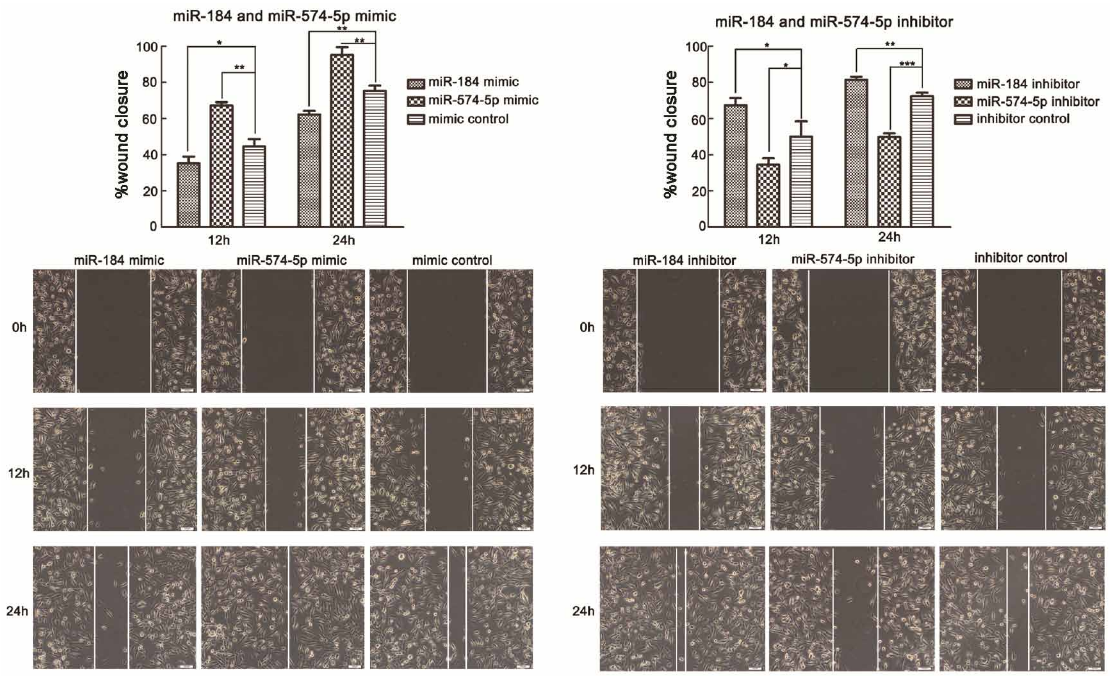

Supplementary Figure S2: miR-574-5p promoted the wound healing process in H446 cells, whereas miR-184 suppressed it. Upper panel, wound healing results for H446 cells transfected with the indicated mimics or inhibitors (analyzed by *t*-test). Lower panel, pictures of wounds of H446 cells transfected with the indicated mimics or inhibitors, taken at 0, 12 and 24 h after the wounds were inflicted (×40). *, $p < 0.05$; **, $p < 0.01$; ***, $p < 0.001$.

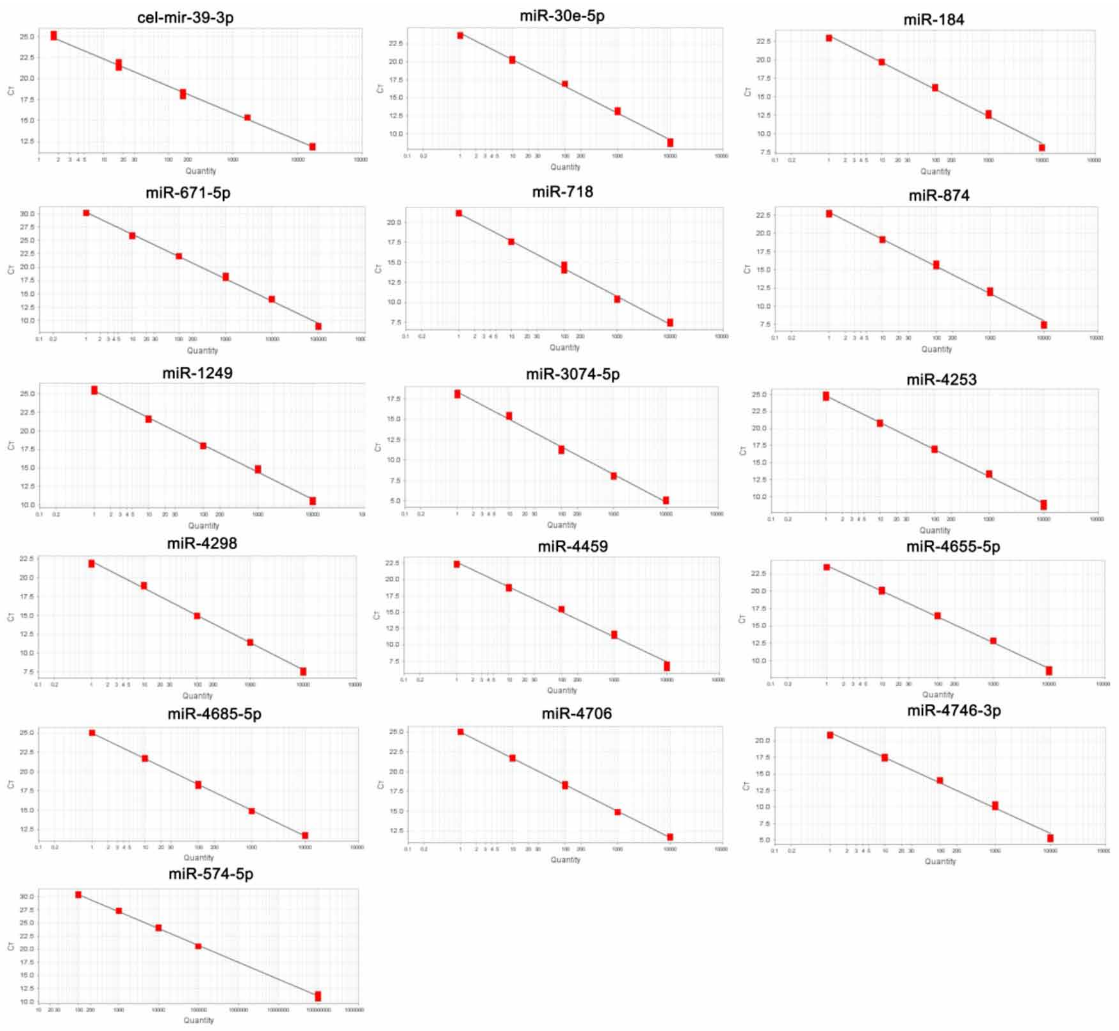

Supplementary Figure S3: Amplification curve for the miRNA primers. All sets of primers had an amplification efficiency of approximately 90–110%.

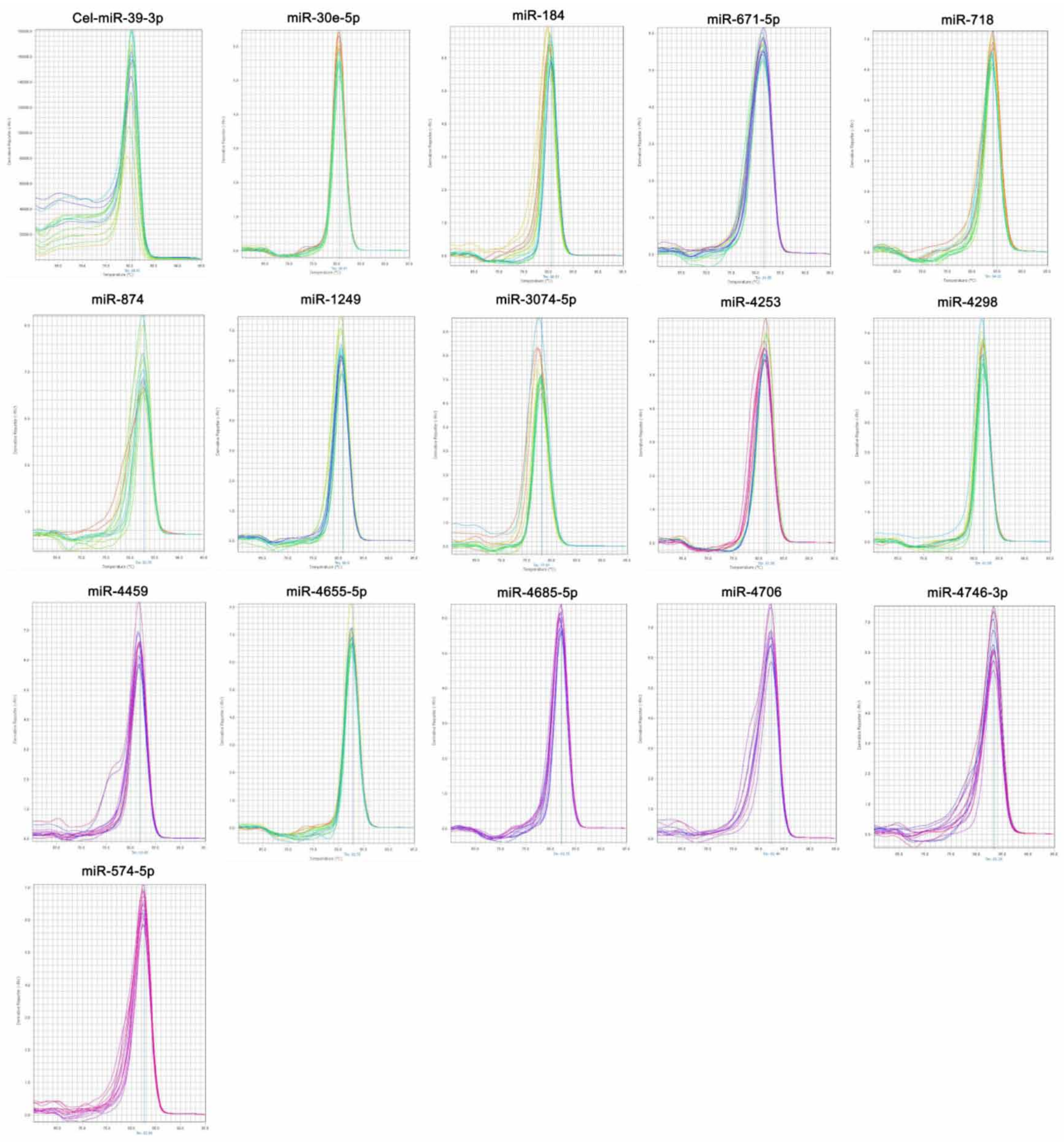

Supplementary Figure S4: Dissolution curves for the miRNA primers. All dissolution curves contained one Tm peak at approximately 80°C.

Supplementary Table S1: Selected characteristics of the SCLC patient population recruited at the cancer center of Wuhan Union Hospital between 2012-11-28 and 2014-6-28

	LD Stage	ED Stage	*p*-value
Characteristic	(*N* = 22)	(*N* = 50)	(LD vs. ED)
Age[a], years			
Mean/Median	58.0/57	57.0/57	0.472
Range	38–75	34–75	
Gender[b], *n*(%)			
Male	18(81.8)	34(68.0)	0.228
Female	4(18.2)	16(32.0)	
Smoking status[b], *n*(%)			
Never	5(22.7)	10(20.0)	0.460
Former	6(27.3)	8(16.0)	
Current	11(50.0)	32(64.0)	
ECOG status[b], *n*(%)			
0	10(45.5)	17(34)	0.355
1	12(54.5)	33(66)	
Metastasis status[b], *n*(%)			
Metastasis	0	46	<0.001
No metastasis	22	4	
MFT, days(range)	511.5(214–798)	212(99–476)	

[a]Used *t*-test
[b]Used Chi-square test
Abbreviations: MFT, median follow-up time. The follow-up time ended on 2015-08-22.

Supplementary Table S2: Selected characteristics of the SCLC patients chosen for miRNA microarray analysis

	**LD Stage**	**ED Stage**	***p*-value**
Characteristic	(*N* = 3)	(*N* = 3)	(LD vs. ED)
Age[a], years			
Mean/Median	62.0/60	63.3/64	0.832
Range	50–72	56–74	
Gender[b], *n*(%)			
Male	3(100)	2(66.7)	1.000
Female	0(0)	1(33.3)	
Smoking status[b], *n*(%)			
Never	1(18.2)	1(15.0)	0.513
Former	1(36.4)	0(35.0)	
Current	1(45.5)	2(50.0)	
ECOG status[b], *n*(%)			
0	3(100)	2(66.7)	0.273
1	0(0)	1(33.3)	
Metastasis status[b], *n*(%)			
Metastasis	0(0)	3(100)	0.014
No metastasis	3(100)	0(0)	

[a]Used *t*-test
[b]Used Chi-square test
Abbreviations: ED, extensive disease; LD, limited disease.

Supplementary Table S3: Differentially expressed miRNAs (*p* <0.05) with at least a two-fold change, as obtained from comparisons of sera from ED and LD participants

Expression in ED		Fold Change	*p*-value	Genomic Location
Up	1 hsa-miR-4685-5p	84.47765	8.12E-05	chr10
	2 hsa-miR-4746-3p	84.34494	0.000114	chr19
	3 hsa-miR-3074-5p	64.37504	0.000211	chr9
	4 hsa-miR-30e-5p	20.14565	0.03968	chr1
	5 hsa-miR-874	18.903013	0.038156	chr5
	6 hsa-miR-574-5p	2.4744165	0.014834	chr4
Down	1 hsa-miR-4706	411.5068	0.000134	chr14
	2 hsa-miR-184	166.55504	0.000915	chr15
	3 hsa-miR-4253	147.18361	0.038342	chr1
	4 hsa-miR-4655-5p	68.21347	0.039199	chr7
	5 hsa-miR-4298	4.2707477	0.032606	chr11
	6 hsa-miR-671-5p	3.9945033	0.026079	chr7
	7 hsa-miR-4459	3.5370402	0.029699	chr5
	8 hsa-miR-4738-3p	3.3797996	0.007289	chr17
	9 hsa-miR-718	3.0091803	0.010574	chrX
	10 hsa-miR-1249	2.3383913	0.04697	chr22
	11 hsa-miR-5585-3p	2.0009031	0.012573	chr1

Abbreviations: ED, extensive disease; LD, limited disease

Supplementary Table S4: Serum miRNAs significantly correlated with the SCLC stage

	Mean Rank		Mann-Whitney *U* test
**No. samples**	**50**	**22**	**Sig.**
**miRNA name**	**Extensive Disease**	**Limited Disease**	
**hsa-mir574-5p**	44.46	18.41	<0.001***
**hsa-mir874**	43.56	20.45	<0.001***
**hsa-mir3074-5p**	43.02	21.68	<0.001***
**hsa-mir4685-5p**	42.48	22.91	<0.001***
**hsa-mir4746-3p**	40.88	26.55	0.007**
hsa-mir184	31.09	48.80	0.001**
hsa-mir4459	33.02	44.41	0.033*

NOTE: **Bold** indicates high expression in the extensive disease stage.
**p*-value < 0.05;
***p*-value < 0.01;
****p*-value < 0.001

Supplementary Table S5: Selected characteristics of the SCLC patients from whom both serum and tissue were collected

	**LD Stage**	**ED Stage**	***p*-value**
Characteristic	(*N* = 15)	(*N* = 30)	(LD vs. ED)
Age[a], years			
Mean/Median	57.5/56	56.97/57.5	0.766
Range	38–74	34–75	
Gender[b], *n*(%)			
Male	13(86.7)	21(70)	0.220
Female	2(13.3)	9(30)	
Smoking status[b], *n*(%)			
Never	5(33.3)	6(20.0)	0.241
Former	5(33.3)	6(20.0)	
Current	5(33.3)	18(60.0)	
ECOG status[b], *n*(%)			
0	6(40)	10(33.3)	0.660
1	9(60)	20(66.7)	
Metastasis status[b], *n*(%)			
Metastasis	0(0)	30(100)	<0.001
No metastasis	15(100)	0(0)	

[a]Used *t*-test
[b]Used Chi-square test
Abbreviations: ED, extensive disease; LD, limited disease.

Supplementary Table S6: Tissue miRNAs significantly correlated with the SCLC stage

	**Mean Rank**		**Mann-Whitney *U* test**
**No. samples**	**30**	**15**	**Sig.**
**miRNA name**	**Extensive Disease**	**Limited Disease**	
**hsa-mir574-5p**	30.10	8.80	<0.001***
**hsa-mir4746-3p**	28.20	12.60	<0.001***
hsa-mir184	17.80	33.40	< 0.003**
hsa-mir4459	18.83	31.33	0.033*

NOTE: **Bold** indicates high expression in the extensive disease stage.
*p-value < 0.05;
**p-value < 0.01;
**p-value < 0.001

Supplementary Table S7: The TaqMan stem-loop primers for reverse transcription PCR and the forward and reverse primers for real-time PCR

cel-miR-39-3p	Sequence	UCACCGGUGUAAAUCAGCUUG
	TaqMan primer	GTCGTATCCAGTGCAGGGTCCGAGGTATTCGCACTGGACGACCAAGCT
	PCR-F	TCCGTCTCCGGGTGTAAATCAG
	PCR-R	CAGTGCAGGGTCCGAGGTAT
hsa-miR-718	Sequence	GGCCGCGGCGCGCAAGAUGGCGGCGGGCCCGGGCACCG CCCCUUCCGCCCCGCCGGGCGUCGCACGAGGC
	TaqMan primer	GTCGTATCCAGTGCAGGGTCCGAGGTATTCGCACTGGATCGACCGAGC
	PCR-F	ATATTCTTCCGCCCCGCC
	PCR-R	CAGTGCAGGGTCCGAGGTAT
hsa-miR-1249	Sequence	GGGAGGAGGGAGGAGAUGGGCCAAGUUCCCUCUGGC UGGAACGCCCUUCCCCCCCUUCUUCACCUG
	TaqMan primer	GTCGTATCCGTGCGGGTCCGAGGTATTCGCACTGGATACGACTGAAGA
	PCR-F	AAATGCTAACGCCCTTCCCC
	PCR-R	CAGTGCAGGGTCCGAGGTAT
hsa-miR-4685-5p	Sequence	CCCAGGGCUUGGAGUGGGGCAAGGUU
	TaqMan primer	GTCGTATCCAGTGCAGGGTCCGAGGTAT TCGCACTGGATACGACAACCT
	PCR-F	TTACCCAGGGCTTGGAGTG
	PCR-R	CAGTGCAGGGTCCGAGGTAT
hsa-miR-4746-3p	Sequence	AGCGGUGCUCCUGCGGGCCGA
	TaqMan primer	GTCGTATCCAGTGCAGGGTCCGAGGTAT TCGCACTGGATACGACTCGGCC
	PCR-F	ATTAAGCGGTGCTCCTGCG
	PCR-R	CAGTGCAGGGTCCGAGGTAT
hsa-miR-30e-5p	Sequence	UGUAAACAUCCUUGACUGGAAG
	TaqMan primer	GTCGTATCCAGTGCAGGGTCCGAGGTAT TCGCACTGGATACGACCTTCCA
	PCR-F	GCCGCTGTAAACATCCTTGAC
	PCR-R	CAGTGCAGGGTCCGAGGTAT
hsa-miR-3074-5p	Sequence	GUUCCUGCUGAACUGAGCCAG
	TaqMan primer	GTCGTATCCAGTGCAGGGTCCGAGGTAT TCGCACTGGATACGACCTGGCT
	PCR-F	GCACGTTCCTGCTGAACTGA
	PCR-R	CAGTGCAGGGTCCGAGGTAT
hsa-miR-874	Sequence	CUGCCCUGGCCCGAGGGACCGA
	TaqMan primer	GTCGTATCCAGTGCAGGGTCCGAGGTAT TCGCACTGGATACGACTCGGTC
	PCR-F	ATTACTGCCCTGGCCCGA
	PCR-R	CAGTGCAGGGTCCGAGGTAT

(*Continued*)

hsa-miR-4706	Sequence	AGCGGGGAGGAAGUGGGCGCUGCUU
	TaqMan primer	GTCGTATCCAGTGCAGGGTCCGAGGTAT TCGCACTGGATACGACAAGCAG
	PCR-F	ATTAAGCGGGGAGGAAGTGG
	PCR-R	CAGTGCAGGGTCCGAGGTAT
hsa-miR-4253	Sequence	AGGGCAUGUCCAGGGGGU
	TaqMan primer	GTCGTATCCAGTGCAGGGTCCGAGGTA TTCGCACTGGATACGCACCCCC
	PCR-F	ACACAGAGGGCATGTCCAGG
	PCR-R	CAGTGCAGGGTCCGAGGTAT
hsa-miR-4655-5p	Sequence	CACCGGGGAUGGCAGAGGGUCG
	TaqMan primer	GTCGTATCCAGTGCAGGGTCCGAGGTAT TCGCACTGGATACGACCGACCC
	PCR-F	AATAACACCGGGGATGGCAGA
	PCR-R	CAGTGCAGGGTCCGAGGTAT
hsa-miR-4298	Sequence	CUGGGACAGGAGGAGGAGGCAG
	TaqMan primer	GTCGTATCCAGTGCAGGGTCCGAGGTAT TCGCACTGGATACGACCTGCCT
	PCR-F	AAGACTGGGACAGGAGGAGG
	PCR-R	CAGTGCAGGGTCCGAGGTAT
hsa-miR-4459	Sequence	CCAGGAGGCGGAGGAGGUGGAG
	TaqMan primer	GTCGTATCCAGTGCAGGGTCCGAGGTAT TCGCACTGGATACGACCTCCAC
	PCR-F	TTAACCAGGAGGCGGAGGAG
	PCR-R	CAGTGCAGGGTCCGAGGTAT
hsa-miR-184	Sequence	UGGACGGAGAACUGAUAAGGGU
	TaqMan primer	GTCGTATCCAGTGCAGGGTCCGAGGTAT TCGCACTGGATACGACACCCTT
	PCR-F	GCGTTGGACGGAGAACTGAT
	PCR-R	CAGTGCAGGGTCCGAGGTAT
hsa-miR-671-5p	Sequence	AGGAAGCCCUGGAGGGGCUGGAG
hsa-miR-574-5p	Sequence	UGAGUGUGUGUGUGUGAGUGUGU

*The primer sets for hsa-miR-671-5p (ssD089261711, ssD090525045, ssD809230631, MQP-0101) and hsa-miR-574-5p (ssD089261711, ssD809230531, ssD809231223, MQP-0101) were purchased form RiboBio, Guangdong, China. The sequences of the primer sets for hsa-miR-671-5p and hsa-miR-574-5p belong to the same commercial company (RiboBio, Guangdong, China) and are not currently publicly available

Supplementary Sequence S1: Sequence of wild and mutant EPAS1 mRNA 3′UTR. The underlined part is the insertion sequence. The mutant sites are at 209 bp (missing 3 Ts), 520 bp (A changed to C), and 727 bp (one T added). GGAGGAGCTCCAGATGAATGGGTAAGTACATCAAGAGCTTCGTGGAGCGCGTGCTGAAGAACGAGCA
GTAATTCTAGGCGATCGCTCGAGCAACTGTCCATACTAACAAGTTTGGTGCATGTCTGTTCTTCTGTAGGGA
GAAGCTTTAGCTTCATTTTACTAAAAAGATTCCTCGTTATTGTTGTTGCCAAAGAGAAACAAAAA
TGATTTTGCTTTCCAAGCTTGGTTTGTGGCGTCTCCCTCGCAGAGCCCTTCTCGTTTCTTTTTTAAACTAATCACCATA
TTGTAAATTTCAGGGTTTTTTTTTTTTTGTTTAAGCTGACTCTTTGCTCTAATTTTGGAAAAAAAAGAAATGTGAAGGGTC
AACTCCAACGTATGTGGTTATCTGTGAAAGTTGCACAGCGTGGCTTTTCCTAAACTGGTGTTTTTCCCCCGCATTTGG
TGGATTTTTTATTATTATTCAAAAACATAACTGAGTTTTTTAAAAGAGGAGAAAATTTATATCTGGGTTAAGTGTTTAT
CATATATATGGGTACTTTGTAATATCTAAAAACTTAGAAACGGAAATGGAATCCTGCTCACAAAATCACTTTAAGATCT
TTTCGAAGCTGTTAATTTTTCTTAGTGTTGTGGACACTGCAGACTT
GTCCAGTGCTCCCACGGCCTGTACGGACACTGTGGAAGGCCTCCCTCTGTCGGC
TTTTTGCCATCTGTGATATGCCATAGGTGTGACAATCCGAGCAGTGGAGTCATTC
AGCGGGAGCACTGCGCGCTATCCCCTCACATTCTCTATGTACTATGTATGTATGTATTATTATTAT
TGCTGCCAAGAGGGTCTGATGGCACGTTGTGGGGTCGGGGGGTGGGGCGGGGAAGTGCTCTAACTTTT
CTTAAGGTTTTGTTGCTAGCCCTTCAAGTGCACTGAGCTATGTGACTCGGATGGTCTTTCACACGGCAC
ATTTGGACATTTCCAGAACTACCATGAGATGGTTTAGACGGGAATTCATGCAAATGAGGGGTCAAAAAT
GGTATAGTGACCCCGTCCACGTCCTCCAAGCTCACGACCTTGGAGCCCCGTGGAGCTGGACTGAGGAGG
AGGCTGCACAGCGGGAGAGCAGCTGGTCCAGACCAGCCCTGCAGCCCCCACTCAGCCGGCAGCCAGAT
GGCCCCGCAAGGCCTCCAGGGATGGCCCCTAGCCACAGGCCCTGGCTGAGGTCTCTGGGTCGGTCAGTG
ACATGTAGGTAGGAAGCACTGAAAATAGTGTTCCCAGAG
CACTTTGCAACTCCCTGGGTAAGAGGGACGACACCTCTGGTTTTTC
AATACCAATTACATGGAACTTTTCTGTAATGGGTACAATGAAGAAGT
TTCTAAAAACACACACAAAGCACATTGGGCCAACTATTTAGTAAGCCCGGATAG
ACTTATTGCCAAAAACAAAAAATAGCTTTCAAAAGAAATTTAAGTTCTATGAGAAATTCCTTAGTCATGGTGTTG
CGTAAATCATATTTTAGCTGCACGGCATTACCCCACACAGG
GTGGCAGAACTTGAAGGGTTACTGACGTGTAAATGCTGGTATTTGAG
CGGCCGCTGGCCGCAATAAAATATCTTTATTTCATTACATCTGTGTGTGGT

Supplementary Sequence S2: Sequence of wild and mutant PTPRU mRNA 3′UTR. The underlined part is the insertion sequence. The mutant sites are CACACTCA (28~35 bp) changed to GTGTGAGT and CACACTC (95~101 bp) changed to GTGTGAG. GGAGGACGCTCCAGATGAATGGGTAAGTACATCAAGAGCTTCGTGGAGCGCGTG
CTGAAGAACGAGCAGTAATTCTAGGCGA TCGCTCGAGAGATAGCGGGGCCCTGGCCTGGGGCACCCACTGCACACTCAGG
GCCAGACCCACCATCCTGGACTGGCGAGGAAGATCAGTGCCTCCTGCTCTGCCCAAACACACTCCCATG
GGGCAAGCACTGGAGTGGATGCTGGGCTATCTTGCTCCCCCTTCCACTGTGGGCAGGGCCTTTCGCTTGT
CCCATGGGCGGGTGGTGGGCCAAGGAGGAGCTTAGCAAGTCTGCAGCCCAGCCCCACCTCCATAGGGTC
CTGCAGGCCTGTGCTGAGAGGCCTGGTGCTGCCTGGCAGAGTGACAAAGGCTCAGGACGGCTGGCTCTG
GGGGACTCAGGCCAAGCCCCTTGGCACCATCCTGGCTTTTGGCAGGGATGAGTGAGGCCCTGCAGAGA
GCATCCCAGGCCAAGGTTCCCACTCAGCCTGCCCCCTCTGCATGTGGGTAGAGGATGTACTGGGACTTG
GCATTTAGGATTCCATCTGGCCCAGCCCCTGAAGGTCCTGGGGAAGCAGGTCTCAATTCTGAATAGCCA
GTGGGGCACACTGACTGTCCTCCCCAGGGGAACTGCAGCGCCCTCCTCCCCACTGCCCCCTGCAGCCC
CTGAGATATTTTGCTCACTATCCCTCCCCACTTGCTTCCCTGATATGTGCTCTGAGCTTCCCTGAACCAG
GATCTGCCTATTACTGCTGTGCCCCATGGGGGGCTCCTTCCCTGCCTGACCCACTGTTGCAGAATGAAG
TCACCTCGCCCCCCTCTTCCTTTAATCTTCAGGCCTCACTGGCCTGTCCTGCTCAGCTTGGGCCAGTGAC
AATCTGCAAGGCTGAACAACAGCCCCTGGGGTTGAGGCCCCTGTGGCTCCTGGTCAGGCTGCCCGTTGT
GGGGAGGGGCAGTGTTAGAGCAGGGCTGGTCATACCCTCTGGAGTTCAGAGGAAGAGGTAGGACCAGT
GCTTTTTTGTTTCTTTTGTTATTTTTGGTTGGGTGGGTGGGAAGGTCTCTTTAAAATGGGGCAGGCCAC
ACCCCCATTCCGTGCCTCAATTTCCCCATCTGTAAACTGTAGATATGACTACTGACCTACCTCGCAGGGG
GCTGTGGGGAGGCATAAGCTGATGTTTGTAAAGCGCTTTGTAAATAAACGTGCTCTCTGAATGCCACAGGCGGCCGCTGGC
CGCAATAAAATATCTTTATTTCATTACATCTGTGTGTGTT
